# Deep learning method for comet segmentation and comet assay image analysis

**DOI:** 10.1038/s41598-020-75592-7

**Published:** 2020-11-03

**Authors:** Yiyu Hong, Hyo-Jeong Han, Hannah Lee, Donghwan Lee, Junsu Ko, Zhen-yu Hong, Ji-Young Lee, Ju-Hyung Seok, Hee Seon Lim, Woo-Chan Son, Insuk Sohn

**Affiliations:** 1 Department of R&D Center, Arontier Co., Ltd, Seoul, Republic of Korea; 2 Department of Medical Science, Asan Medical Institute of Convergence Science and Technology, University of Ulsan College of Medicine, Asan Medical Center, 88 Olympic-ro 43-gil, Songpa-gu, Seoul, Republic of Korea; 3 Asan Institute of Life Sciences, Asan Medical Center, 88 Olympic-ro 43-gil, Songpa-gu, Seoul, Republic of Korea; 4 Department of Pathology, University of Ulsan College of Medicine, Asan Medical Center, 88 Olympic-ro 43-gil, Songpa-gu, Seoul, 05505 Republic of Korea

**Keywords:** Computational biology and bioinformatics, Drug discovery

## Abstract

Comet assay is a widely used method, especially in the field of genotoxicity, to quantify and measure DNA damage visually at the level of individual cells with high sensitivity and efficiency. Generally, computer programs are used to analyze comet assay output images following two main steps. First, each comet region must be located and segmented, and next, it is scored using common metrics (e.g., tail length and tail moment). Currently, most studies on comet assay image analysis have adopted hand-crafted features rather than the recent and effective deep learning (DL) methods. In this paper, however, we propose a DL-based baseline method, called DeepComet, for comet segmentation. Furthermore, we created a trainable and testable comet assay image dataset that contains 1037 comet assay images with 8271 manually annotated comet objects. From the comet segmentation test results with the proposed dataset, the DeepComet achieves high average precision (AP), which is an essential metric in image segmentation and detection tasks. A comparative analysis was performed between the DeepComet and the state-of-the-arts automatic comet segmentation programs on the dataset. Besides, we found that the DeepComet records high correlations with a commercial comet analysis tool, which suggests that the DeepComet is suitable for practical application.

## Introduction

DNA damage is a major toxicological concern in drug development as it is a critical cause of cancer, as well as chronic and degenerative diseases such as heart diseases and Parkinson’s disease^1–6^. The DNA can be damaged by spontaneous chemical reactions, reactive oxygen, and nitrogen species generated by metabolism, or exogenous agents. If a damaged DNA is not repaired correctly, the damages might be accumulated and lead to mutations, cell death, or senescence that can induce various diseases including cancers and chronic diseases^1^. The assessment of DNA damage, therefore, is crucial in the toxicology field to detect genotoxicity and carcinogenic potential of certain compounds. It is also significant in medical research to understand the pathogenesis of diseases including cancer and chronic diseases, and can be applied in clinical medicine to monitor these disease conditions^7,8^.

Although there are several methods to assess DNA damage^9–12^, comet assay (also referred to as single-cell gel electrophoresis) is recommended for detecting the DNA strand breakage in ICH S2 (R1) guideline^13^. Its high sensitivity, high efficiency, and low cost made it easily accessible for researchers^14,15^. In the assay, damaged DNA migrates out of the nucleus to form a shape that resembles a tail of a comet during electrophoresis, whereas undamaged DNA in the nucleus forms the head of the comet shape. Followed by electrophoresis, DNA is visualized by staining with DNA-binding dye under fluorescence microscopy. If the comet head gets smaller and dimmer while its tail gets longer and brighter, it suggests that the DNA in the cell is significantly damaged. Hence, each cell detected in the image is referred to as a comet.

The damage of DNA on comet assay images can be determined either by visual scoring or computer image analysis^16^. Visual scoring classifies comets into several different categories based on DNA damage degree (visually estimated tail length and shape) subjectively. However, computer image analysis provides a range of different parameters of each comet by pixel-based computation. Although the visual scoring is simple and may appeal to those who cannot afford expensive computer programs, the computer image analysis is preferable in assessing DNA damage as it can provide objective quantified information of comets.

Several image analysis methods, such as Comet Score, CASP, OpenComet, CometQ, and HiComet for comet assay have been proposed to facilitate the comet scoring process^16–20^. They have two main stages, i.e., comet detection or segmentation and comet scoring. Depending on whether detection or segmentation process gets completed automatically, it can be divided into either a semi- or fully-automated tool. The primary performance differences come from whether a tool can detect and segment each comet correctly rather than scoring stage because the metric used to assess the comet score has predefined basic standards and rules^21^. However, these image analysis methods all use hand-crafted image features with traditional machine learning (such as support vector machine) at comet detection and segmentation stage. Users should define features manually and tune visually which might be laborious. It makes the methods have a few limitations on capturing comets from a noisy background and hardly distinguish two individual cells when overlapped. In contrast, DL can allow raw image data as input and learn to detect and segment comet in an end-to-end process.

The DL provides excellent achievements in the fields of computer vision, such as image classification^22^, image object detection^23,24^, and image object segmentation^25–27^. The DL outperforms hand-crafted features by 10%–20%^28^. Typically, the performance advantage is based on two aspects. One is the development of effective convolutional neural network (CNN) methods and the enormous processing capacity of modern hardware. Next is the availability of large, well-organized, and publicly accessible image datasets, such as MNIST^29^, ImageNet^30^, and COCO^31^. Although there are a few reports applying deep learning on visual scoring automatically^32–34^, still none of the open-source software tools for comet assay analysis deployed DL methods to detect and segment comets so far. We believe that one of the primary reasons is the lack of suitable datasets to train a deep neural network.

In this paper, a new fully-automated comet assay analysis program (DeepComet) using a DL method (rather than hand-crafted features) is proposed to improve the effectiveness of comet image analysis. A trainable and testable comet assay image dataset with annotations is introduced along with the DL method used to segment comets as well as the overall pipeline of the comet scoring and analysis procedure. Then, the performance of the proposed DL-based baseline method (DeepComet) compared to those of state-of-the-art comet segmentation programs such as OpenComet, HiComet, Comet Assay IV is discussed.

## Materials and methods

### Ethical statement

Canine peripheral blood mononuclear cells (PBMCs) were obtained from six beagle dogs. The use of animals was approved by the Institutional Animal Care and Use Committee of Asan Medical Center (IACUC No. 2019-12-187). All experiments were performed in accordance with relevant guidelines and regulations.

### Image acquisition

To create datasets for a DL model, a comet assay with canine PBMCs was performed. The PBMCs were isolated from whole blood by density centrifugation using Ficoll-Paque Plus, followed by a comet assay directly or cryopreservation. The comet images in these datasets were obtained as follows: 1 × 10^5^ cells/ml PBMCs suspended in the PBS were mixed with low-melting agarose at 37 °C at a ratio of 1:10 (v/v). The mixture was spread onto CometSlide (Trevigen Inc., Maryland, US). After solidification of the agarose, the slides were immersed in a lysis solution overnight at 4 °C. They were then transferred into alkaline unwinding solution containing 200 mM NaOH and 1 mM EDTA (pH 13) and incubated for 20 min for DNA unwinding and alkali-labile damage. An electrical field (300 mA, 21 V) was applied for 23 min at room temperature (RT). The slides were washed twice in distilled water for five minutes at RT and then washed once in 70% ethanol for 10 min at RT. After drying, the slides were stained with 100 μL of 1X SYBR Gold (Thermo Fisher Scientific) and captured the comet images using a Nikon inverted microscope.

### Annotation on comets

Two biology researchers who were experienced in the comet assay for over half a year under the guidance of a toxicology expert in the assay annotated the images independently and compared each other’s work to ensure consistency. Here, the VGG Image Annotator^35^ software was used to annotate the images manually. We annotated the intact comet heads without a tail using a circular region tool that outputs the center and radius of the circle. Further, comets with a tail and DNA damage were annotated with a polyline tool, where the first dot was marked at the center of a comet head, and the second dot was marked at the bottom of the comet head. These two points were used to identify the head of a comet. Subsequently, dots were marked along the boundary of the comet tail counterclockwise from the second dot. The total number of dots were at least eight per each comet.

Comets can be sorted into non-ghost cells with a distinct head and tail, and ghost cells with a small or no nucleoid head and a broad tail^36^. The ghost cells are usually classified as the highest degree of DNA damage by visual scoring. Until now, there is a controversy over the cause of ghost cells and how they should be analyzed appropriately^37^. Therefore, our method is designed to classify these two kinds of cells, so one can use some custom analysis techniques for ghost cells.

**Figure 1:**
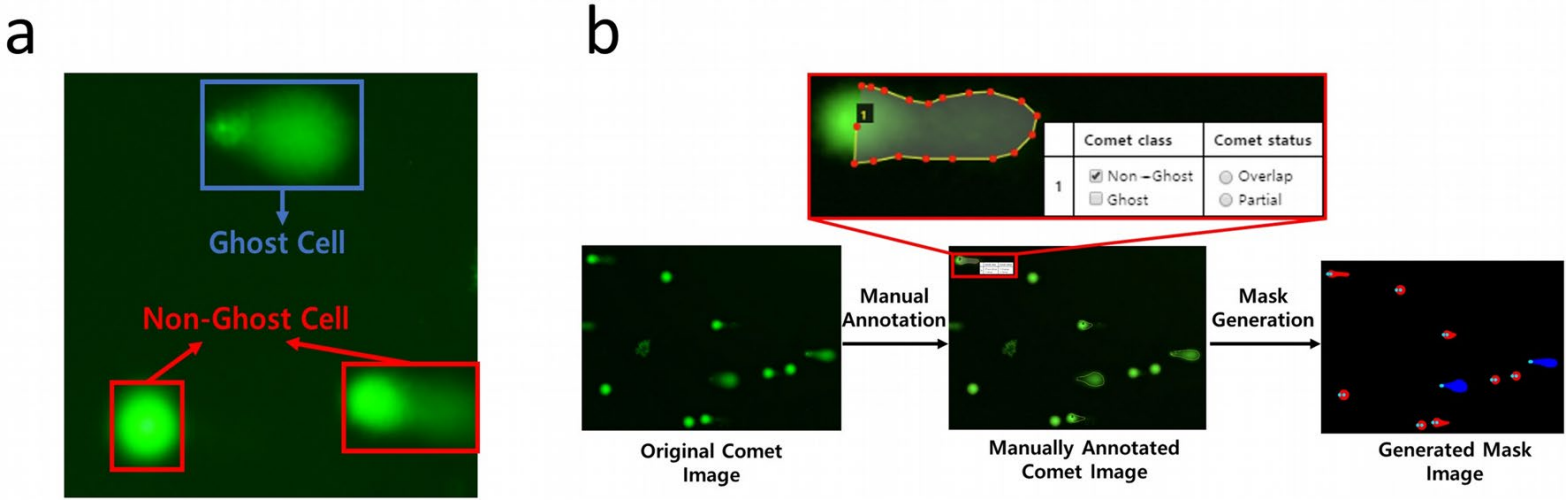
Classification, annotation, and mask generation of cells on comet images**.** (**a**) Non-ghost cells with a distinct head and tail (red), and ghost cells with a small head and a broad tail (blue) are indicated. (**b**) After manual annotation for the boundary, class, and status, each comet is masked with red or blue.

The comets in our datasets were all classified as non-ghost or ghost cells (Fig. 1a). Furthermore, we assigned tags to the comets if they were overlapped or located at the boundary of an image (i.e., only a part of the comet is visible). Figure 1 shows the procedure to generate the ground truth comet mask images to train the DL model. After manual annotation with dots and tags (in the red box), each dotted contour was filled up to give mask images. As shown in Fig. 1b, the non-ghost cell is red, the ghost cell is blue, and the two cyan points in each comet mark the head of the comet in a circular shape.

### Datasets and image variability

**Figure 2:**
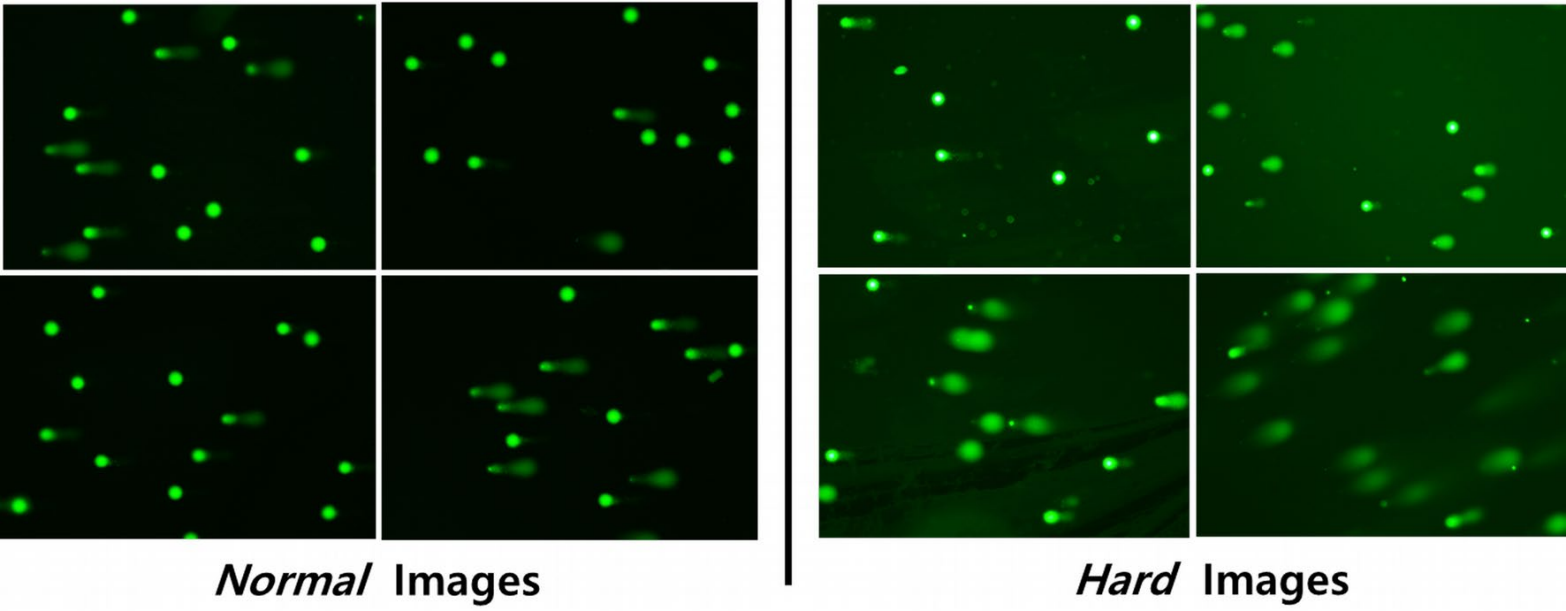
Examples from the *Normal* and *Hard* datasets. The Normal dataset contains comet images with a clean background, whereas the Hard dataset contains comet images with a noisy background, or images in which comet tails are heading in different directions.

To compare DeepComet to the state-of-the-art methods and demonstrate its advantages, we deliberately collected comet images of different qualities. The images were divided based on the quality, into two sub-datasets, i.e., the *Normal* and *Hard* datasets (Fig. 2). The *Normal* dataset contains comet images with a clean background, which are preferred for analysis, and was used to test the state-of-the-art software^16–20^. However, the *Hard* dataset contains comet images with a noisy background, which makes it challenging to identify individual comets or images in which comet tails are heading in different directions. The noise in the *Hard* images could be due to agarose gel not drying adequately, phase difference caused by gel thickness, or dust in the gel.

Table 1 shows the characteristics of the comet image dataset. All images were 2048 × 2880 pixels, the total number of images was 1037 (i.e., with 8271 comet objects; 5787 non-ghost cells, and 2484 ghost cells), and the average number of comets in each image is 7.98. The *Normal* and *Hard* datasets had 589 and 448 images with 4552 and 3719 comets, respectively. The number of non-ghost cells was approximately twice the number of ghost cells. The ratio between non-ghost and ghost cells was much higher in the *Normal* dataset compared to the *Hard* dataset, i.e., there were many ghost cells in the *Hard* dataset.

**Table 1:** Characteristics of datasets for comet segmentation.

Dataset	Image size	Number of images	Number of objects			Averaged number of objects in image		
			Non-ghost	Ghost	Total	Non-ghost	Ghost	Total
*Normal*	2048 $\times$ 2880	589	3588	964	4552	6.09	1.64	7.73
*Hard*		448	2199	1520	3719	4.91	3.39	8.30
Total		1037	5787	2484	8271	5.58	2.40	7.98

Comet image could vary in comet shape and pixel intensity and image resolution depending on sample materials, experimental setup, or laboratory environments such as dust, equipment, or electricity. Consequently, the comet images made from different laboratories can be different enough to result in the bias of each comet segmentation method^16–20^ against data. Since it is difficult to obtain external comet image datasets from other laboratories, each comet segmentation method might be optimized for their own comet images from a single laboratory. Previously reported comparison results of comet analysis programs^16,34^ have shown a large gap in performance between their developed method and others when experimented with using their own dataset, which indicates the image variability issue. Given that there is no publicly available well-organized comet assay image dataset, we released our dataset to be utilized by researchers for fairer comparison between methods.

### Deep learning

#### Comet segmentation

Mask R-CNN, a DL model, was used as a baseline for the comet segmentation task and implemented the model using a well-documented open-source code (https://github.com/facebookresearch/maskrcnn-benchmark, https://github.com/pytorch/vision/blob/master/torchvision/models/detection/mask_rcnn.py). Also, the Pytorch framework was utilized to implement the Mask R-CNN.

The Mask R-CNN comprises of four main modules: the backbone, region proposal network (RPN), region of interest (ROI) classifier and bounding box regressor, and mask segmentation modules. The backbone module is a feature extractor that takes images as input and outputs feature maps. Notice that the backbone module can be any deep CNN proposed for image analysis. The RPN module is a small CNN that runs on the feature maps and scans them using a sliding window to find ROIs, i.e., bounding boxes with a high probability of containing objects but not a background. The ROI classifier and bounding box regressor module is also a small CNN that works on the ROIs from the RPN. The ROI classifier examines and classifies each ROI as specific classes, and it identifies whether an ROI is a non-ghost or ghost cell. The bounding box regressor further refines the size and location of the bounding box from the RPN module. Finally, the mask segmentation module is a small, fully convolutional NN used to produce the desired object segmentation masks for each positive region selected by the ROI classifier and bounding box regressor module. It is a binary mask indicating the pixels of the object in the bounding box.

Thus, each non-ghost cell was separated into a head and tail to be further characterized by parameters, such as the percentage of DNA in the head and tail. The mask segmentation module segmented the total occupied region of the comet. The Mask R-CNN was extended by adding a comet head segmentation module to predict and utilize two key points. The first point is the center point of the head, and the other is the leftmost point of the head boundary, as we considered the head as a circle region. The training target gets defined by viewing each key point as a one-hot binary mask, where only a single pixel is labeled as the foreground. The comet head segmentation module predicts two masks, one for each of the two key point types (i.e., the head center and leftmost point).

The following configurations and hyperparameters of Mask R-CNN were used for training and testing. The feature pyramid network with Resnet-50^38,39^ was selected as the backbone. All training and test images were resized from 2048 $\times$ 2880 to 512 $\times$ 720, and then a batch size of four was used to input images to the Mask R-CNN model. The model was initialized with weights pre-trained using the MSCOCO dataset^31^. The number of output classes was two (i.e., the non-ghost and ghost cells). In total, the model was trained for 20 epochs using a stochastic gradient descent optimizer with a momentum of 0.9, weight decay of 0.0005, and an initial learning rate of 0.005, which was reduced by a factor of 10 in every five epochs. Notice that the RPN and box regression modules have many other minor hyperparameters. Here, the default settings of the original Mask R-CNN was applied, except that the non-maximum suppression in RPN and bounding box regressor was set to 0.5 and 0.2, respectively, in the testing stage to obtain better detection performance. Also, image augmentation techniques were used to improve the robustness of the model. It included the vertical and horizontal translation of the images by random pixel [-10, 10], rotation by arbitrary degree [− 30, 30], and random vertical flip. To mitigate variations between the comet assay images caused by different experimental environments and exposure extent during scanning, random brightness modulation [− 0.25, 0.25] and contrast modulation [0.25, 1.75] were performed as well.

#### Comet scoring

**Figure 3:**
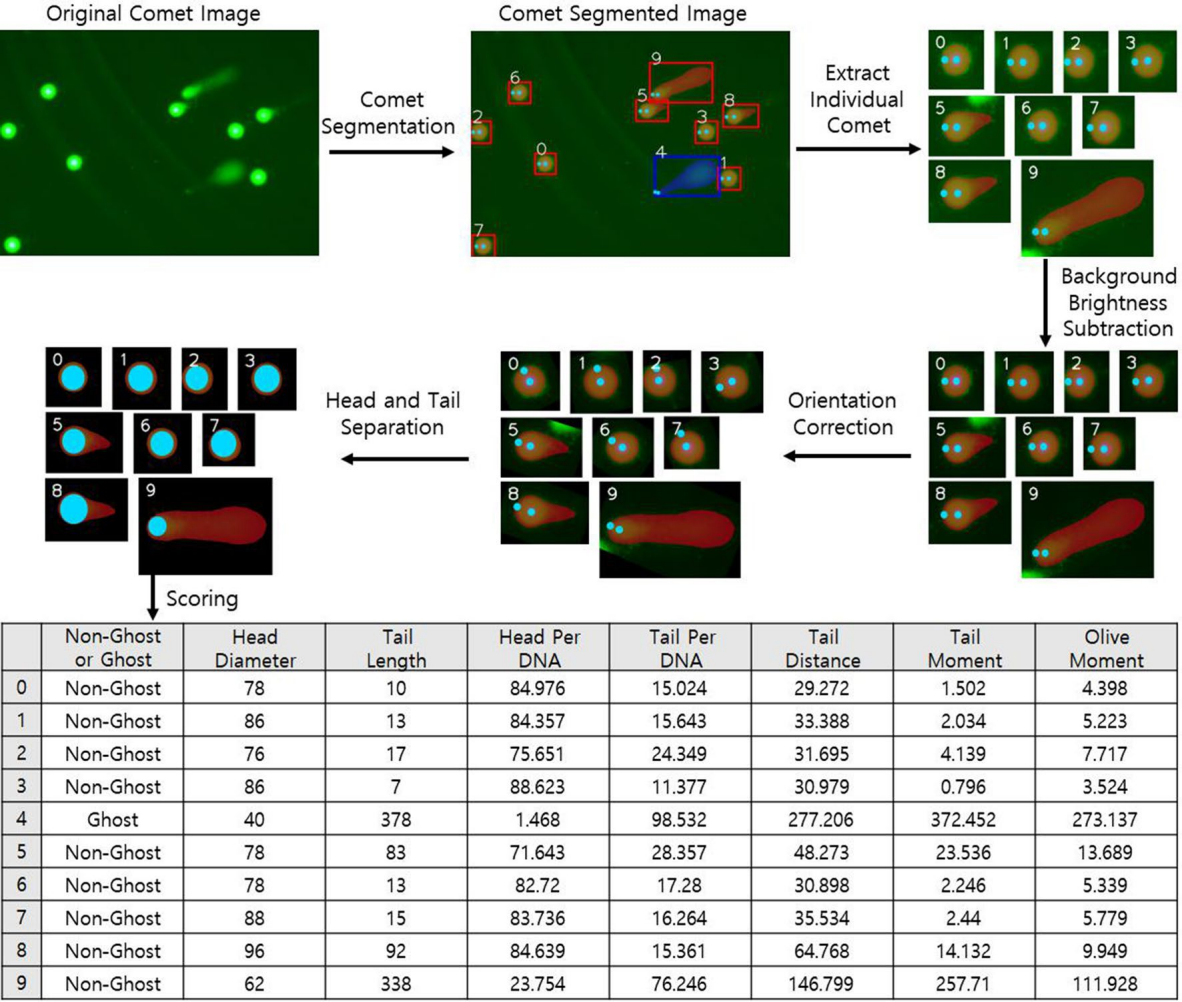
Workflow of comet scoring procedure. First, each comet is extracted with its bounding box. If the background brightness is irregular, it is subtracted from the comet intensity. If the tails are not horizontal, the orientation is corrected for all the comets. Then, the head and tail are separated using the two predicted key points of the comet head, marked by a circled cyan mask. Finally, comets are scored based on the parameters, as shown in the sample table.

After segmentation, the comets are processed as follows (Fig. 3). First, an individually segmented comet is extracted with its bounding box. Then, in some cases, background illumination is spread irregularly over the image; thus, the brightness of the local background was subtracted from the comet intensity to improve the effectiveness of the score measurement. Here, the local background brightness is calculated by selecting the median intensity along with a 15-pixel wide contour area of the predicted comet bounding box. Next, the comet tail sometimes extends in a different direction for various reasons; therefore, the orientation is corrected to avoid erroneous calculation of the comet scores. The comet rotation angle is calculated by fitting an ellipse on the segmented comet mask region, and the angle between the x-axis and the major axis of the ellipse is calculated. The head and tail are separated using the two predicted critical points of the comet head. Finally, various parameters are calculated based on previous studies^14,21,40^ and a guideline^22^ suggests that the amount of DNA in a region is proportional to the integrated intensity values of the pixels in that region. Three comet scores demonstrated effectiveness in measuring DNA damage, i.e., the percentage of the DNA in the tail (DNA (%) in the tail), tail moment (tail length times percentage of DNA in the tail), and the Olive moment (distance between the centers of the head and tail times the rate of DNA in the tail)^41,42^. We output these comet scores with additional scores concerning DNA damage, which are listed in the example table in Fig. 3.

## Results

### Training and testing the deep learning model with datasets

**Figure 4:**
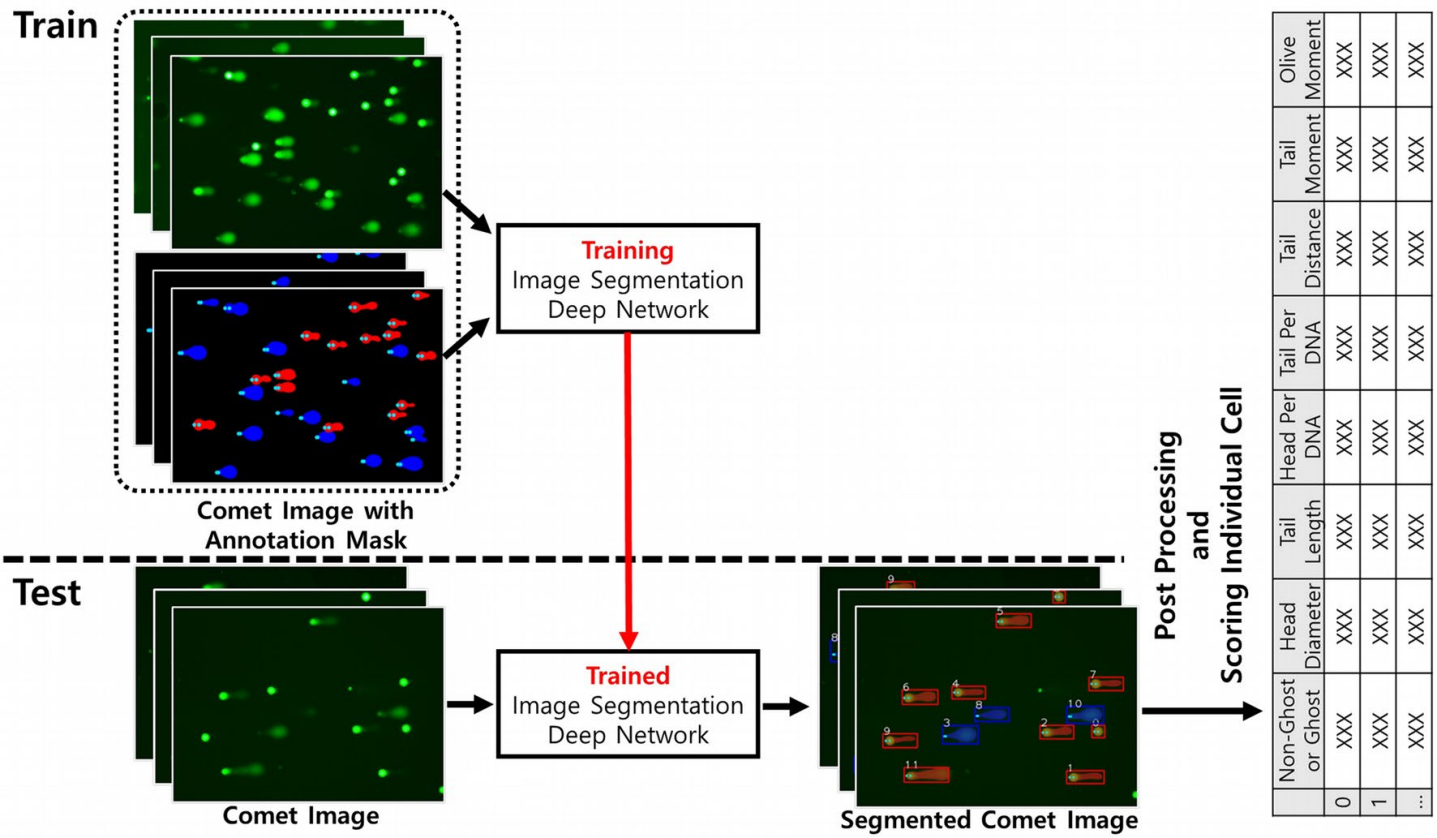
Overview of the proposed framework for comet analysis. In the training stage, a deep neural network is trained using a comet image and a corresponding mask image to enable the model to precisely segment each comet in the image. Besides, it enables the classification of the ghost and non-ghost cells. In the testing stage, the trained comet segmentation model is first used to segment and classify each comet. Then, followed by applying a series of post processing techniques, a general comet scoring method is applied to score them and place the results together to generate a result table for each comet image.

100 images were randomly selected from the *Normal* and *Hard* datasets for testing. The remaining images were used to train the Mask-RCNN model. Consequently, the training dataset had 837 images (i.e., 489 *Normal* and 348 *Hard* images) containing 6690 comet objects comprising 4725 non-ghost and 1965 ghost cells. The test dataset had 200 images containing 1581 comet objects comprising 1062 non-ghost and 519 ghost cells. Figure 4 shows an overview of the proposed framework, including the training and testing stages.

### Comet segmentation performance

The comet segmentation performance was evaluated using the precision/recall curve and average precision (AP)^43^ to measure the accuracy of image instance segmentation and image object detection. Here we give a brief explanation of these metrics, which were used in computer vision tasks.

#### Intersection over Union (IoU)

**Figure 5:**
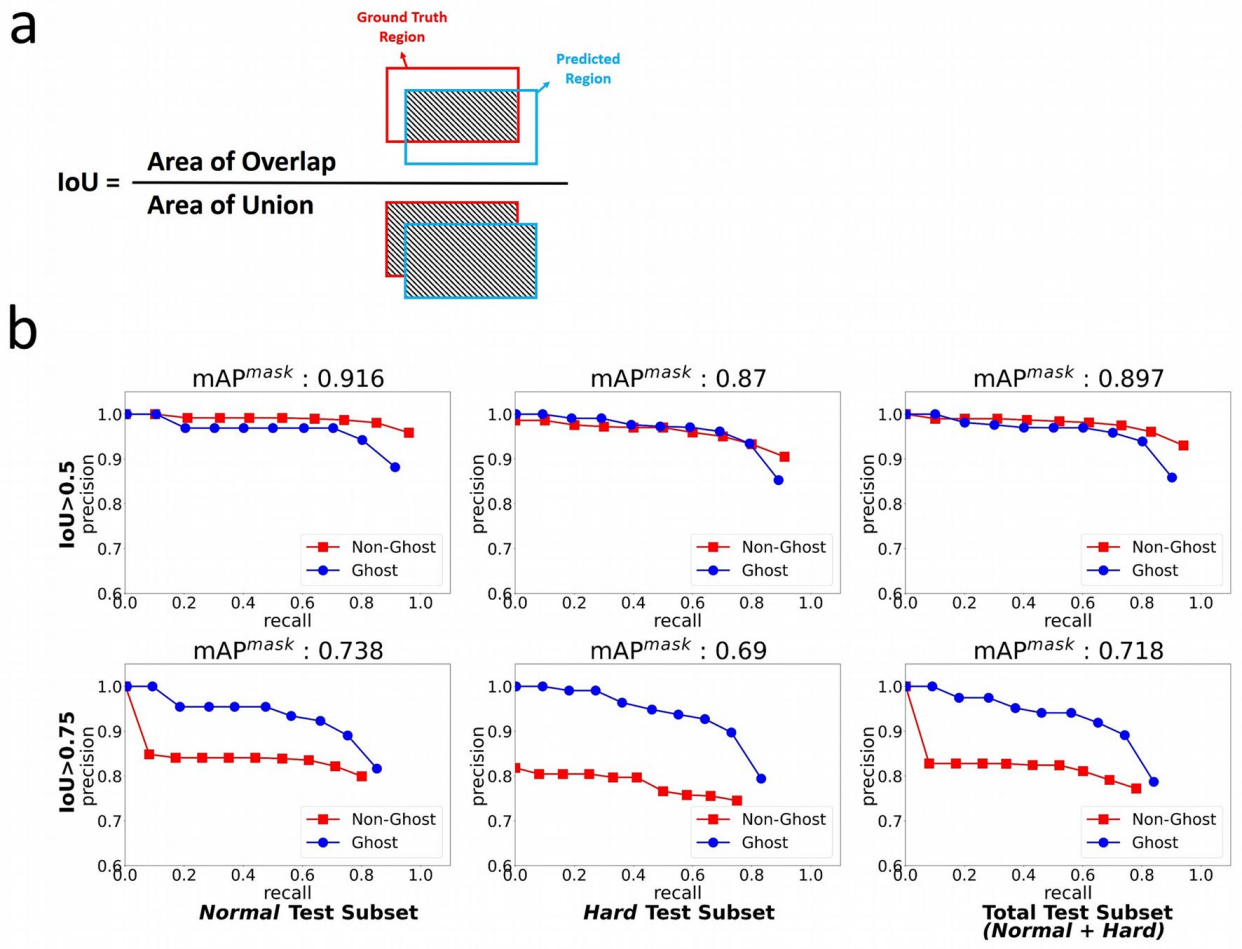
Precision/recall curves of the DeepComet on the test dataset. (**a**) Intersection over Union (IoU) is given by the overlapping area between the predicted region and the ground truth region divided by the area of union of them. (**b**) The precision/recall curve of the DeepComet at IoU > 0.5 and IoU > 0.75 on our test dataset. The precision/recall curve can be used to evaluate the performance of an object detection model.

The IoU measures the overlap between the two detected regions, which can be bounding boxes or segmentation masks. It is given by the overlapping area between the predicted region and the ground truth region divided by the area of the union (Fig. 5a). The terms involved are explained as follows:

#### True positive (TP)

A detection where IoU ≥ threshold and the predicted class agrees with the ground truth class.

#### False positive (FP)

An incorrect detection.

#### False negative (FN)

A ground truth object not detected.

#### Precision

It measures relevant objects among all detected ones; it is expressed as follows.

1 $$Precision = \frac{TP}{{TP + FP}}$$

#### Recall

It measures detected objects among all relevant ones; it is expressed as follows:

2 $$Recall = \frac{TP}{{TP + FN}}$$

The AP is the precision averaged across all recall values between 0 and 1 by varying the detection confidence threshold. The COCO metric uses 101 interpolated data points to calculate the AP. In the following, AP^mask^ denotes that the IoU was estimated between segmentation masks, AP^bb^ indicates that the IoU was calculated between bounding boxes, mAP is the mean AP over all classes, AP@0.5 denotes that the IoU threshold was 0.5, and AP@[0.5:0.95] indicates that the AP was averaged over 10 IoU thresholds (from 0.5 to 0.95 with a step size of 0.05).

Figure 5b shows the precision/recall curve of the DeepComet at IoU > 0.5 and IoU > 0.75 on our test dataset. The precision/recall curve can be used to evaluate the performance of an object detection model as the confidence is changed by plotting a curve for each object class. A model is considered reliable if the curve stays close to y (precision) = 1, which means that the accuracy remains high as recall increases by varying the confidence threshold. For the segmentation of non-ghost and ghost cells, the DeepComet demonstrated that its precision remained over 0.9 until recall increased to approximately 0.8 at IoU > 0.5. The mAP^mask^@0.5 on the *Normal* test subset was 0.916, which is slightly higher than that of the *Hard* test dataset (0.87) and the total (*Normal* + *Hard*) test dataset (0.897). The results obtained on the *Hard* test dataset indicate that the DeepComet was well trained for segmenting comets on these noisy images. At IoU > 0.75, although the curves dropped as expected, the precision values were more significant than 0.8 until recall increased to approximately 0.8. The exception was when the recall value was over 0.4 with the *Hard* test dataset, where the precision values were slightly less than 0.8. Unlike at IoU > 0.5, there were gaps between the non-ghost curve and the ghost curve when IoU > 0.75.

In Table 2, we evaluated AP^mask^ and mAP^mask^ averaged over IoU thresholds from 0.5 to 0.95 with a step size of 0.05. The mAP^mask^@[0.5:0.95] values of the *Normal, Hard*, and total datasets were 0.616, 0.584, and 0.601, respectively. In general, the DeepComet demonstrated a better performance in segmenting ghost cells compared to non-ghost cells on two datasets.

**Table 2:** Comet segmentation performance of DeepComet on test dataset by AP^mask^@[0.5:0.95] and mAP^mask^@[0.5:0.95].

Dataset	Non-Ghost AP^mask^@[0.5:0.95]	Ghost AP^mask^@[0.5:0.95]	mAP^mask^@[0.5:0.95]
*Normal*	0.589	0.643	0.616
*Hard*	0.538	0.63	0.584
Total (*Normal* + *Hard*)	0.569	0.633	0.601

Table 3 compares the performance of the DeepComet with OpenComet Version.1.3.1 (https://www.cometbio.org)^17^ and HiComet (https://github.com/taehoonlee/HiComet)^19^. We applied default settings in each program. Since these two programs have no function to classify cells into non-ghost and ghost cells, we ignored the classification function of the DeepComet and simply transformed the output as detection of comets for comparison. The outputs of the HiComet are the detected bounding boxes of the comets. Thus, here the AP^bb^ metric was used to evaluate and compare the results. As shown in Table 3, the DeepComet showed much higher AP^bb^ values in all set-ups. The AP^bb^@0.5 of our results were all greater than 0.9 on the three test datasets, whereas the results obtained using the OpenComet and HiComet were under 0.5. Regarding the high IoU threshold, the AP^bb^@0.75 and AP^bb^@[0.5:0.95] values of the DeepComet were approximately 0.78 and 0.66, respectively, whereas the compared programs barely reached 0.2. When comparing the corresponding AP^bb^ results obtained by the *Normal* and *Hard* test datasets, it was demonstrated that the gap between the results from each dataset was much less for the DeepComet compared to other methods, where DeepComet showed a difference of less than 5% but greater than 30% for others. However, it should be considered that the experimental setup can be unfavorable for OpenComet and HiComet as they were not optimized for our comet image dataset.

**Table 3:** Comet object bounding box detection performance by AP^bb^. OpenComet and HiComet were operated with default settings.

Program	*Normal*			*Hard*			*Normal* + *Hard*		
	AP^bb^@ 0.5	AP^bb^@ 0.75	AP^bb^@ [0.5:0.95]	AP^bb^@ 0.5	AP^bb^@ 0.75	AP^bb^@ [0.5:0.95]	AP^bb^@ 0.5	AP^bb^@ 0.75	AP^bb^@ [0.5:0.95]
OpenComet	0.446	0.117	0.178	0.290	0.065	0.111	0.359	0.08	0.137
HiComet	0.382	0.1	0.162	0.169	0.049	0.066	0.255	0.067	0.105
DeepComet	0.960	0.771	0.665	0.920	0.788	0.664	0.942	0.781	0.664

**Figure 6:**
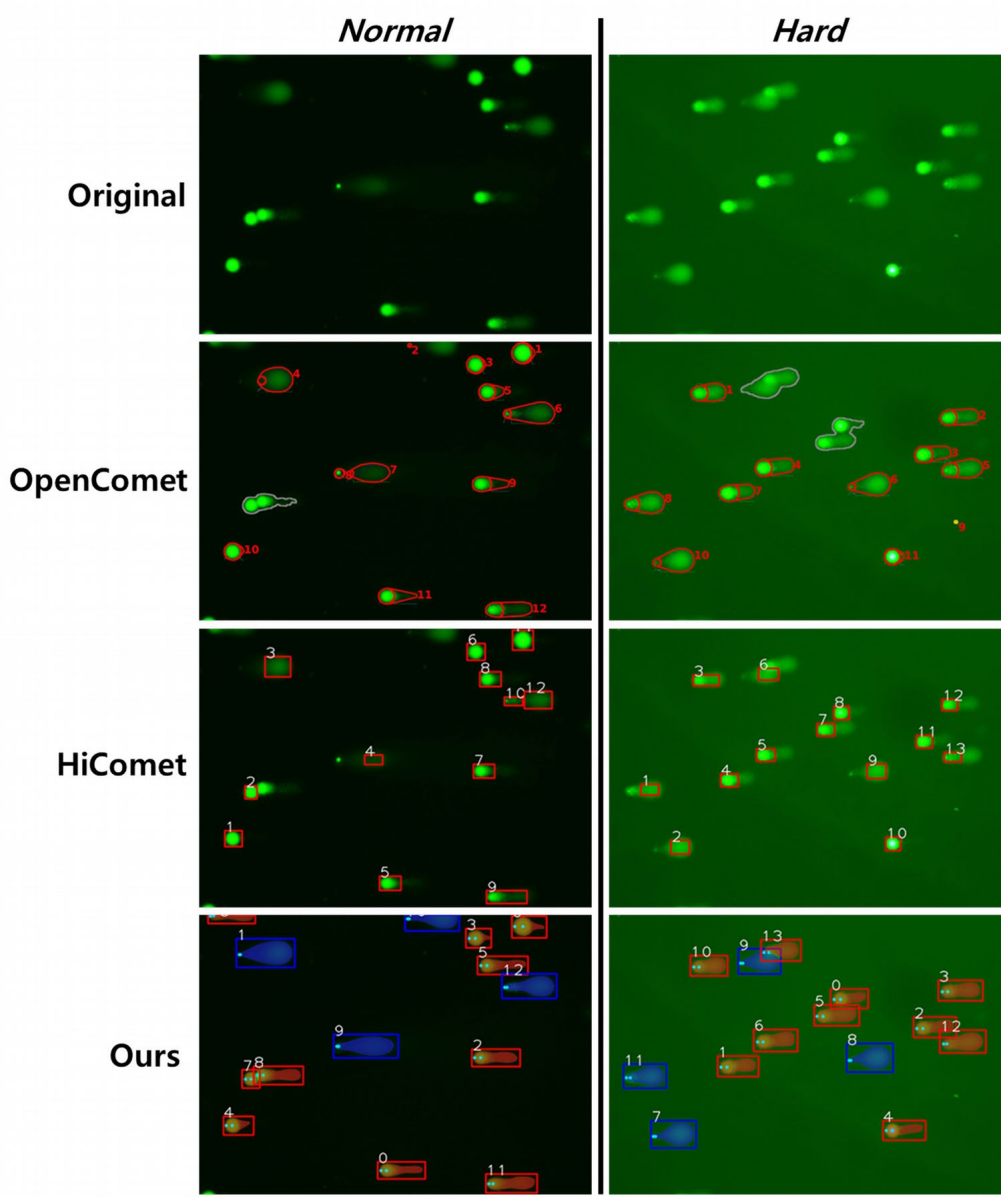
The comparison of comet segmentation results from the DeepComet and other automatic programs.

**Figure 7:**
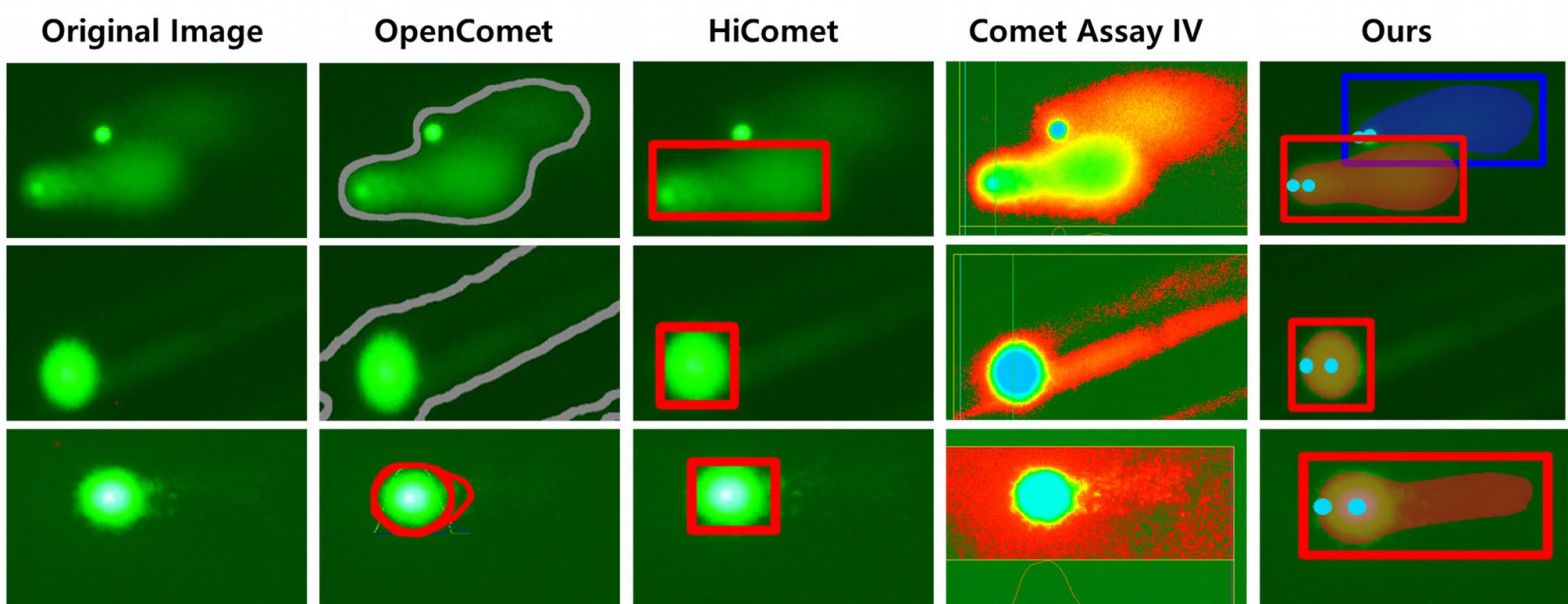
The comparison of individual comet segmentation results from the DeepComet and other programs.

Figures 6 and 7 show the visual comparisons of the comet detection and segmentation results. Figure 6 shows the representative images from the *Normal* and *Hard* test datasets for comparison. The OpenComet could not segment slightly overlapped targets into individual comets. Furthermore, it did not effectively detect the entire tail of the comet for the segmentation No. 5 in the *Normal* image and No. 11 in the *Hard* image. There were also incorrect segmentations for ghost cells. From the HiComet output images in the third row, it was found that HiComet did not adequately recognize the tail part of the non-ghost cell and could not capture the entire ghost cell. However, the DeepComet in the bottom was able to segment slightly overlapped comets into two individuals. Also, the DeepComet captured a dim tail in the *Normal* image and a noisy tail in the *Hard* image. The DeepComet could also distinguish non-ghost and ghost cells correctly, and capture objects even on the edge of the image.

Figure 7 shows the segmentation results on representative comets, which prove challenging to segment due to overlapping, background noise, or a dim tail (bright background). Here, we added the results from Comet Assay IV Version 4.3 (Instem-Perceptive Instruments Ltd., Suffolk, Halstead, UK), which is a semi-automated method that requires a manual click on a comet to score. As shown in the first row, if two comets slightly overlap each other, the OpenComet and Comet Assay IV could not separate them and recognize each comet. In contrast, the HiComet only knew one of the comets. The second row shows the results on an image with background noise. Here, OpenComet never recognized the comet as valid, and Comet Assay IV detected the comet but could not segment it accurately, whereas the HiComet did it correctly. The images in the bottom have a bright background and a dim comet tail. The OpenComet and HiComet did not capture the entire tail, and the Comet Assay IV captured excessive area, including the background.

### Comet score comparison

**Figure 8:**
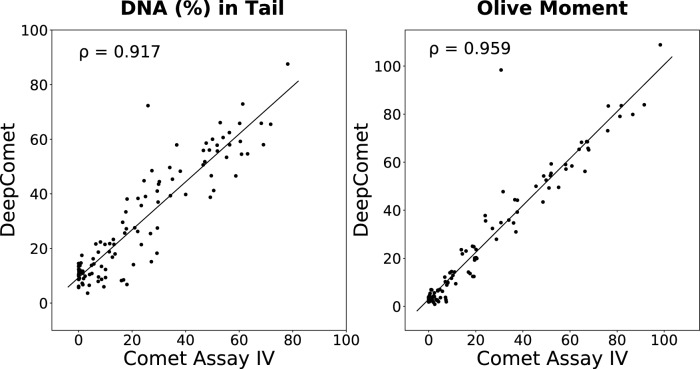
Comet score correlations between DeepComet and Comet Assay IV. For both parameters, DNA (%) in tail and Olive moment, the correlation was positive and significantly high.

We validated our calculated comet scores by verifying the correlation with those from Comet Assay IV using randomly selected 107 non-ghost cells that could easily be segmented. The two most crucial comet parameters, DNA (%) in tail and Olive moment, were calculated, and the correlation between the scores obtained from the DeepComet and Comet Assay IV were analyzed using Pearson correlation. Figure 8 shows the correlations between the scores obtained for DeepComet and Comet Assay IV to be 0.917 for DNA (%) in tail and 0.959 for Olive moment. For both scores, the correlation was positive and significantly high.

**Figure 9:**
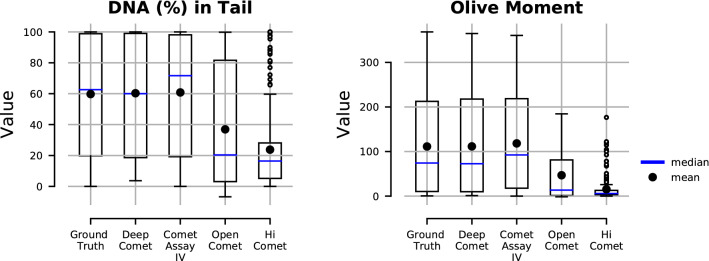
Comet score distributions evaluated at full comet assay image level. For both parameters, DNA (%) in tail and Olive moment, the result from DeepComet was the closest to the ground truth, followed by Comet Assay IV. OpenComet and HiComet were operated with default settings.

In practical application, a fully-automated comet image analysis program should input sets of full comet assay images and automatically output all detected and segmented comets with their scores. Here, the comet scoring performance of the DeepComet was validated in comparison with Ground truth, Comet Assay IV, OpenComet, and HiComet. Figure 9 shows box plots of comet score (DNA (%) in tail and Olive moment) distributions of 50 comet assay images which were randomly selected from *Normal* and *Hard* test subsets (25 for each). The ground truth comet score distribution was calculated on manually annotated comet regions. The comet scores using Comet Assay IV were generated by manual click on every comet on the images using the program. For the objects that Comet Assay IV did not segment properly, we manually modified the comet regions as accurately as possible. False positive detection of comets which had no overlapping region with ground truth were all excluded. It is observed from the box plots that the results of our method match the ground truth the most, followed by Comet Assay IV which also shows quite similar shapes of box plots to the ground truth. OpenComet and HiComet detected almost all comets, however, the segmented comet tails were too short on our datasets, whereas Comet Assay IV tended to segment a comet tail a little wider and longer. Student’s t-test was carried out at the significance level of 0.05 to identify statistical difference between Ground truth and respective other methods. There was no significant difference between Ground truth and DeepComet as well as between Ground truth and Comet Assay IV, whereas there was a significant difference between Ground truth and OpenComet as well as between Ground truth and HiComet.

## Discussion and conclusion

In this paper, we proposed DeepComet for comet segmentation that utilizes the Mask R-CNN architecture. Developed by Facebook AI research, the Mask R-CNN is one of the most popular architectures because it can be extended to other applications easily, such as human pose estimation. Its performance also has been verified in various practical applications^44,45^ and competitions (https://www.kaggle.com/competitions, https://github.com/matterport/Mask_RCNN) of image segmentation. Consequently, we selected the Mask R-CNN among different DL models for image object segmentation^25–27^.

Abundant and well-organized datasets with annotations play an essential role in developing and applying DL techniques to improve performance further. Because there was no available dataset for training the DL model, we obtained plenty of images by performing comet assays with PBMCs. To support and validate the proposed DL model, we constructed a trainable and testable comet assay image dataset with 1037 images containing 8271 comet objects that were all manually annotated. The dataset comprises the *Normal* and *Hard* sub-datasets, where the *Hard* dataset includes images with a noisy background, making automatic comet segmentation challenging. To enable more researchers to train and test with their DL methods to improve the quality of comet assay image analysis, we have released the datasets of comet assay images with annotated comet regions and related tags (https://liveuou-my.sharepoint.com/:u:/g/personal/wooec52_liveuou_kr/EVIbAAmwXUBArB0B4uEmREMBe15FZTJB1LdU40KN8wwDUQ?e=i3jcnB).

There has been debate over the cause of ghost cells and how they should be analyzed appropriately. Due to their appearance, measurements of DNA (%) in the tail through image analysis are unreliable^22^. The DeepComet can classify cells into non-ghost and ghost cells, which helps users to apply customized measurements on ghost cells. With further post processing (e.g., background brightness subtraction, orientation correction, and head and tail separation), the comet score can be measured precisely for each segmented comet using the DeepComet.

In the precision/recall curve of DeepComet at IoU > 0.75, the precision values were higher than 0.8 until recall increased to approximately 0.8 except for the *Hard* test dataset, where the precision values were slightly lower than 0.8. Also, there were gaps between the curves of non-ghost and ghost cells. Based on visual observation, this occurred because the area of ghost cells was generally much more significant than non-ghost cells, which made IoU less sensitive to ghost cells. Furthermore, there were several non-ghost cells with very short and dim tails, which led to ambiguous annotations. The other experimental results for non-ghost and ghost cell segmentation demonstrated that the DeepComet obtained high mAP^mask^@0.5 (0.897) with the total test dataset. The results indicate that the DeepComet is superior to state-of-the-art products with comet bounding box detection using AP^bb^. Besides, compared with the *Normal* test dataset, the AP^bb^ of the *Hard* test dataset gets reduced by approximately 30% for the state-of-the-arts, whereas the AP^bb^ of the DeepComet only decreased by less than 5%. In terms of comet scoring, our results proved that the DeepComet has a high correlation with the commercial Comet Assay IV program. Furthermore, the proposed method also shows the high performance when evaluated at a full comet assay image level. These points demonstrate that the DeepComet can be used for practical automatic comet assay image analysis.

Comet image variability of inter-laboratories is inevitable, and it could be a possible reason for the large gap in performance against our dataset between the DeepComet and other methods (as shown in Table 3 and Fig. 9). Comet images from different laboratories could vary in image resolution, background noise, comet shape, brightness, contrast, etc. As each method was developed and optimized using different datasets, DeepComet may also need to be validated on datasets other than ours. There should be datasets released from various places to carry out the more appropriate comparison, as well as to develop a more generalized algorithm.

We demonstrate that our newly proposed DL-based comet assay analysis program, DeepComet, has shown great performance on our publicly released datasets. The DeepComet and our datasets can serve as a baseline for comet image segmentation and analysis to facilitate future research in this field of toxicology and medical science. In the future, we hope to expand the datasets with multiple external sources from different experimental environments and develop a more specific and accurate deep learning-based architecture for comet assay image analysis.

## Data availability

The datasets generated during and/or analyzed during the current study are available here: 10.5281/zenodo.6395303.
