# Author Correction: Deep learning method for comet segmentation and comet assay image analysis

**DOI:** 10.1038/s41598-022-10754-3

**Published:** 2022-04-19

**Authors:** Yiyu Hong, Hyo‑Jeong Han, Hannah Lee, Donghwan Lee, Junsu Ko, Zhen‑yu Hong, Ji‑Young Lee, Ju‑Hyung Seok, Hee Seon Lim, Woo‑Chan Son, Insuk Sohn

**Affiliations:** 1 Department of R&D Center, Arontier Co., Ltd, Seoul, Republic of Korea; 2 Department of Medical Science, Asan Medical Institute of Convergence Science and Technology, University of Ulsan College of Medicine, Asan Medical Center, 88 Olympic‑ro 43‑gil, Songpa‑gu, Seoul, Republic of Korea; 3 Asan Institute of Life Sciences, Asan Medical Center, 88 Olympic‑ro 43‑gil, Songpa‑gu, Seoul, Republic of Korea; 4 Department of Pathology, University of Ulsan College of Medicine, Asan Medical Center, 88 Olympic‑ro 43‑gil, Songpa‑gu, Seoul, 05505 Republic of Korea

Correction to: *Scientific Reports* 10.1038/s41598-020-75592-7, published online 3 November 2020

The original version of the Article contained an error in the Data Availability statement, which included an incorrect link to access the data.

The datasets generated during and/or analyzed during the current study are available in the following domain, https://liveuou-my.sharepoint.com/:u:/g/personal/wooec52_liveuou_kr/EVIbAAmwXUBArB0B4uEmREMBe15FZTJB1LdU40KN8wwDUQ?e=i3jcnB.

now reads:

The datasets generated during and/or analyzed during the current study are available here: 10.5281/zenodo.6395303.

The Article has been corrected.

